# Cuproptosis: a promising new target for breast cancer therapy

**DOI:** 10.1186/s12935-024-03572-2

**Published:** 2024-12-19

**Authors:** Qianqian Jiang, Fei Tong, Yun Xu, Cheng Liu, Qiaoping Xu

**Affiliations:** 1 Department of Pharmacy, Traditional Chinese Medicine Hospital of Changshan, Quzhou, 324200 P.R. China; 2 Department of Clinical Pharmacology, Key Laboratory of Clinical Cancer Pharmacology and Toxicology Research of Zhejiang Province, Cancer Center, Afliated Hangzhou First People’s Hospital, Westlake University School of Medicine, Hangzhou, 310006 China; 3 Department of Pharmacy, Zhujiang Hospital, Southern Medical University, Guangzhou, Guangdong 510280 P.R. China; 4 Department of Pharmacy, Zhejiang Medical&Health Group Hangzhou Hospital, Hangzhou, Zhejiang 310022 China; 5 Department of Pharmacy, The Secend People’s Hospital Of Jiande, Hangzhou, 311604 P.R. China

**Keywords:** Cuproptosis, Breast cancer, Molecular mechanisms, Immunotherapy, Metastasis

## Abstract

Breast cancer (BC) is the leading cause of cancer-related mortality among women globally, affecting approximately one-quarter of all female cancer patients and accounting for one-sixth of cancer-related deaths in women. Despite significant advancements in diagnostic and therapeutic approaches, breast cancer treatment remains challenging due to issues such as recurrence and metastasis. Recently, a novel form of regulated cell death, termed cuproptosis, has been identified. This process disrupts mitochondrial respiration by targeting the copper-dependent cellular pathways. The role of cuproptosis has been extensively investigated in various therapeutic contexts, including chemotherapy, immunotherapy, radiotherapy, and nanotherapy, with the development of novel drugs significantly improving clinical outcomes. This article aims to further elucidate the connection between cuproptosis and breast cancer, focusing on its therapeutic targets, signaling pathways, and potential biomarkers that could enhance treatment strategies. These insights may offer new opportunities for improved patient care and outcomes in breast cancer therapy.

## Introduction

According to GLOBOCAN 2020 statistics, breast cancer has the highest incidence rate among all cancer types. Despite significant advancements in treatment, including the development of tailored therapies for various subtypes such as luminal A, luminal B, HER-2 overexpression, and triple-negative breast cancer (TNBC) [1], conventional treatments like radiation and chemotherapy often damage healthy cells in addition to targeting cancer cells, leading to undesirable side effects. As a result, researchers continue to seek more precise methods to selectively eliminate cancer cells.

**Fig. 1:**
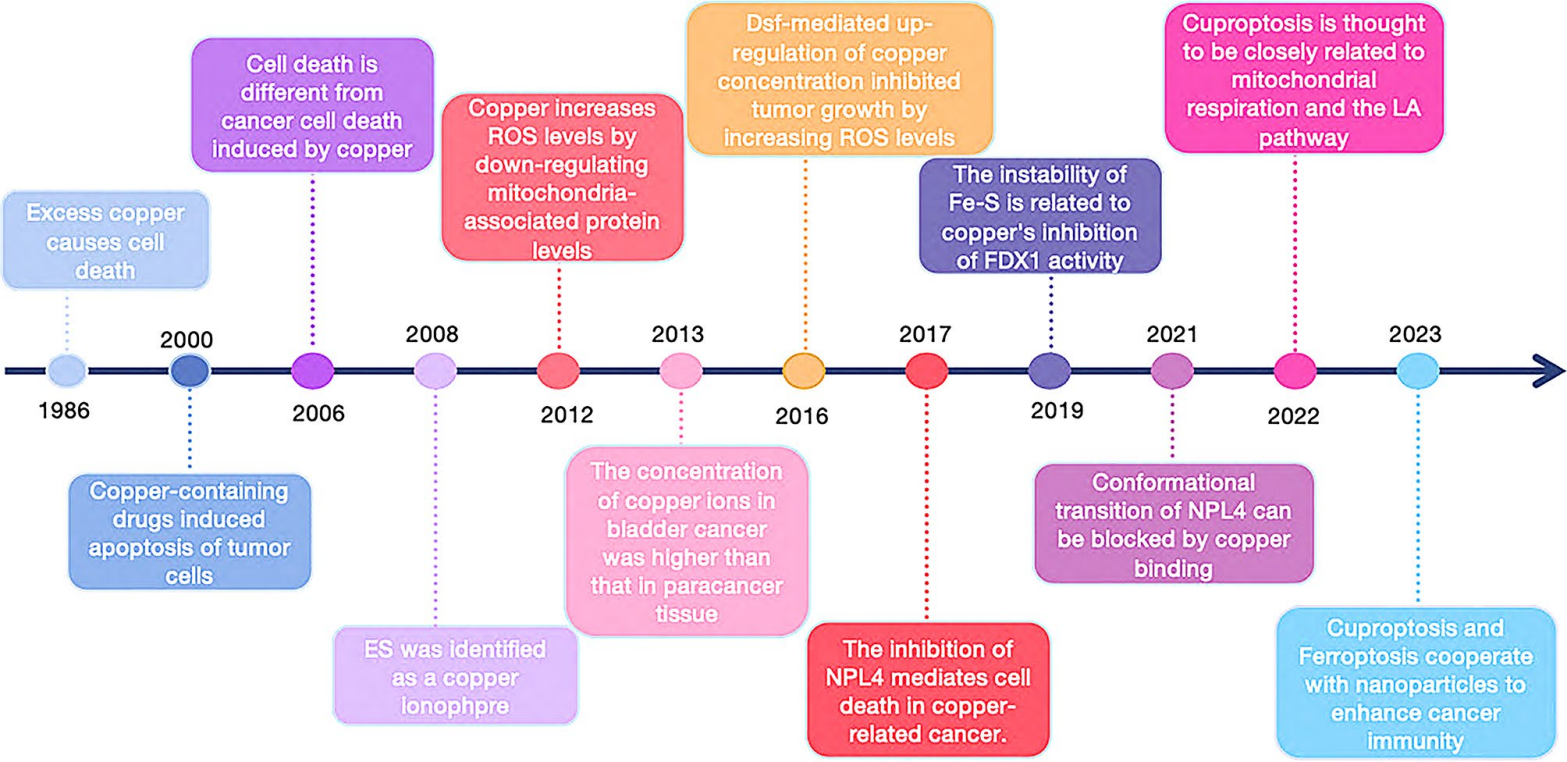
Timeline illustrating the discovery of cuproptosis.Describe on the timeline the historical events that led to the discovery of advances in tumor research that led to cuproptosis and copper-associated cell death

In 2022, Peter Tsvetkov and colleagues introduced the concept of “cuproptosis,” a novel form of cell death induced by copper, which is closely linked to mitochondrial respiration and the lipoic acid (LA) pathway in the human body [2]. Copper ionophores, which have long been explored for their potential antitumor properties, played a key role in the discovery of cuproptosis [3, 4]. As research on copper’s role in cellular processes expands, it is becoming evident that copper-based drugs and technologies interact directly with cancer cells. This review provides a concise overview of recent advances in understanding copper-induced cell death, presenting new opportunities for clinical anti-tumor therapies (Fig. 1). With the growing recognition of cuproptosis, the modulation of cell death via copper regulation is emerging as a promising strategy for breast cancer treatment, offering a strong biomedical foundation for addressing drug resistance in this disease.

### Copper homeostasis and Cuproptosis

Copper is an essential trace element, playing crucial roles in mitochondrial respiration, immune response modulation, and the synthesis of vital biomolecules [5]. It exists in two forms within living organisms: cuprous ions (Cu^+^, the reduced state) and copper ions (Cu^2+^, the oxidized state), both of which are involved in numerous physiological processes. The regulation of copper homeostasis is achieved through a combination of copper intake, utilization, and excretion [6]. Maintaining this delicate balance is critical, as disruptions in copper homeostasis can lead to various health issues. Copper deficiency can result in genetic mutations, neurological disorders, cardiovascular complications, and metabolic disturbances. Conversely, excess copper can lead to copper toxicity, or copper poisoning [7].

To ensure the precise regulation of copper throughout the body, a sophisticated regulatory system is in place. This system relies on duodenal absorption and bile excretion, which work together to maintain copper levels within a healthy range. Copper homeostasis is also controlled at the cellular level, where a complex network of proteins, including copper chaperones, cuproenzymes, and membrane transporters, carefully orchestrates copper regulation. These proteins ensure that copper levels remain within an optimal range, protecting the body from the harmful effects of both copper overload and deficiency, and preserving the balance essential for proper physiological functioning [8].

### Correlation between copper and breast cancer

Cuproptosis, a form of regulated cell death (RCD), is characterized by the direct binding of copper to fatty acylated components of the mitochondrial respiratory tricarboxylic acid (TCA) cycle, inducing protein toxic stress that ultimately leads to cell death. Key cancer-related processes such as angiogenesis, proliferation, growth, and metastasis have been closely linked to copper ion homeostasis. Notably, elevated copper ion concentrations within cancer cells have been identified as a potential marker for cancer progression [9].

Research indicates that patients with malignant tumors exhibit higher levels of copper ions in both their serum and tumor tissues compared to healthy individuals [10]. In breast cancer models, the overload of copper ion carriers has been shown to effectively inhibit tumor growth [11, 12]. A recent meta-analysis, encompassing 36 studies and 4,151 participants, revealed elevated levels of copper (Cu) and cadmium (Cd) in the plasma or serum of breast cancer patients across all biological samples examined [13]. Furthermore, an increased Cu/Zn ratio in both plasma and urine has emerged as an early indicator and risk factor for the development of breast cancer [14]. Elevated levels of copper and copper-related proteins in breast cancer have also been associated with advanced disease stages, tumor microenvironment remodeling, and chemotherapy resistance [15].

Given these findings, copper homeostasis emerges as a valuable marker for monitoring breast cancer progression. Regulating copper concentrations may provide a mechanism for controlling disease progression and metastasis. The modulation of copper levels could induce apoptosis in breast cancer cells, offering promising avenues for treatment. This review consolidates current knowledge regarding the therapeutic potential of copper in breast cancer, emphasizing the importance of maintaining copper homeostasis. The role of copper as a marker for neogenesis and its potential as a therapeutic agent holds significant promise for the development of targeted therapies. By shedding light on these critical aspects, we aim to contribute to the advancement of breast cancer treatment strategies.

### Copper regulation in physiology

Copper is an essential micronutrient, playing a critical role in various physiological processes necessary for human health. It is typically obtained through dietary sources, such as nuts, shellfish, chocolate, seeds, and certain animal products. The recommended daily intake of copper for adults is 0.9 mg [16]. This vital mineral is distributed throughout several organs, including the brain, eyes, liver, and heart [17].

Within the human body, copper exists in two primary forms: cuprous ions (Cu^+^, the reduced state) and copper ions (Cu^2+^, the oxidized form) [18]. Copper is primarily absorbed in the small intestine and then transported via the portal vein to the liver. Once in the liver, serum proteins, predominantly ceruloplasmin (CP) and a minor portion of serum albumin, transport copper throughout the body. Excess copper is stored in the liver and released into systemic circulation as needed. In healthy adults, serum copper concentrations typically range from 70 to 110 mg/dL [19].

Maintaining balanced copper levels is crucial for proper cellular metabolism. Copper deficiency can lead to Menkes disease, a rare disorder marked by developmental abnormalities, neurodegeneration, hypopigmentation, and connective tissue defects [20]. Conversely, excessive copper accumulation in the liver can result in Wilson disease, characterized by liver failure or neurological disorders due to copper buildup in the brain [21]. While these conditions are uncommon, they highlight the critical importance of copper metabolism and the necessity of maintaining appropriate copper ion levels for overall health.

### Copper uptake

Dietary copper is absorbed in the small intestine, where it primarily exists in the extracellular form as Cu^2+^. However, Cu^2+^ cannot directly cross the cell membrane and must first undergo enzymatic reduction. This reduction is facilitated by its binding to the six-transmembrane epithelial antigen of the prostate(STEAP), which converts Cu^2+^ to its reduced form, Cu^+^ [22]. Cu^+^ is then transported across the cell membrane via copper transport protein 1 (CTR1, also known as SLC31A1) [16].

Once inside the cell, Cu^+^ binds to the cytoplasmic copper chaperone for superoxide dismutase (CCS) and superoxide dismutase 1 (SOD1). This complex is subsequently directed to various subcellular compartments, such as the mitochondria, nuclei, and the trans-Golgi network (TGN), where Cu^+^ performs specific functions necessary for cellular metabolism [23].

Current research is increasingly focused on understanding whether these copper transporters, particularly those on the plasma membrane, play a breast cancer-specific role in facilitating copper uptake. Gaining insights into this mechanism could offer valuable understanding of how copper metabolism contributes to breast cancer development and progression.

### Copper utilization

**Fig. 2:**
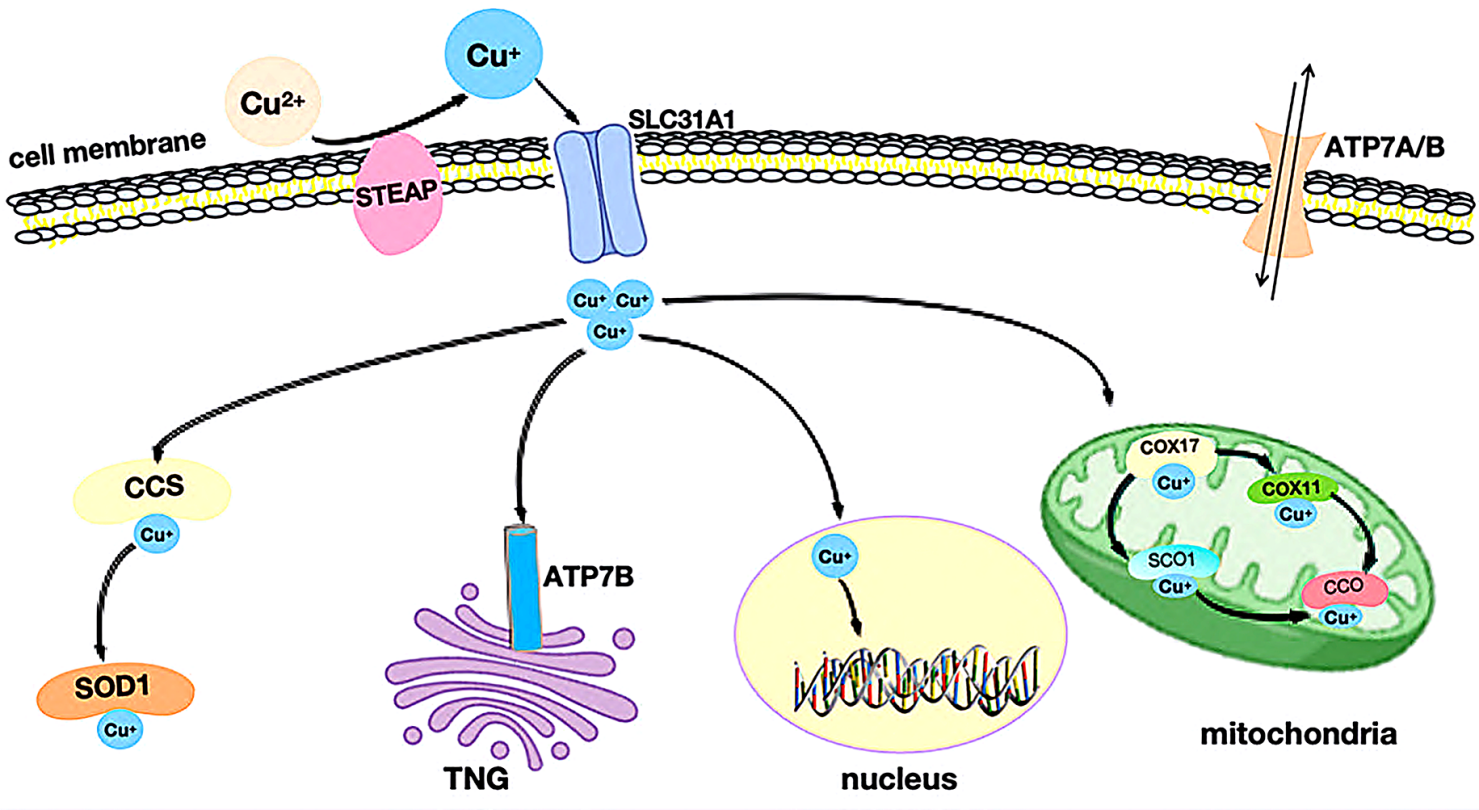
Copper transport in the human body. The absorption of copper ions is regulated by SLC31A1, while ATP7A/B can trigger the efflux of copper. Several copper-binding proteins, such as COX17, and CCS, are responsible for transporting copper to specific subcellular organelles to ensure its bioavailability

Once copper ions enter the cell, they bind to copper chaperones and are directed to specific cellular compartments where they perform distinct functions(Fig. 2). In mitochondria, Cu^+^ combines with cytochrome c oxidase(CCO) and participates in the respiratory chain and redox processes. Cytochrome c oxidase 17(COX17) binds to Cu^+^ in the mitochondrial membrane, transferring it to cytochrome c oxidase 1(SCO1) or cytochrome c oxidase 11(COX11), thus facilitating the incorporation of Cu^+^ into the cytochrome oxidase subunit. Cellular copper pools are essential for mitochondrial oxidative phosphorylation, and the activity of COX17, mitochondrially encoded cytochrome c oxidase I(MT-CO1/COX1), and mitochondrially encoded cytochrome c oxidase II(MT-CO2/COX2) is indispensable. Both MT-CO1 and MT-CO2 play pivotal roles in tumor growth, metastasis, and invasion [24].

In the cytoplasm, CCS (copper chaperone for superoxide dismutase) delivers copper to specific proteins such as SOD1 (superoxide dismutase 1), which plays a functional role in antioxidant defense. While SOD1 is primarily found in cytoplasmic antioxidant proteins, a minor fraction resides in the mitochondrial membrane space. Aberrant SOD1 expression is strongly linked to cancer growth and development [25–27]. Additionally, CCS regulates the positioning of SOD1 between the cytoplasm and membrane space in an oxygen-dependent manner, stabilizing reactive oxygen species (ROS) and preventing oxidative damage caused by copper overload.

In the nucleus, Cu^+^ binds to transcription factors, influencing gene expression. Moreover, Cu^+^ can be transported from the cytoplasm to the lumen of the trans-Golgi network (TGN) by Cu^+^-ATPase transporters, specifically ATPase copper transporters 7 A and 7B (ATP7A/B). These transporters activate copper-dependent enzymes in the secretory pathway. When intracellular Cu^+^ concentrations rise, ATP7A/B relocate from the TGN, leading to Cu^+^ efflux.

The expression of SLC31A1, which regulates copper absorption, is influenced by two key mechanisms. First, the Sp1 transcription factor (SP1) regulates the expression of the SLC31A1 gene [28]. Second, elevated copper levels trigger the phagocytosis and degradation of the SLC31A1 protein [29]. Additionally, copper uptake by solute carrier family 11 member 2 (SLC11A2/DMT1) may act as a compensatory mechanism in cases of SLC31A1 deficiency [30]. Copper ions bind to copper proteins, are distributed across different cellular compartments, and are transported through the portal vein system. Copper is absorbed in the intestinal tract, circulates through the peripheral blood, and eventually reaches the liver, where it is distributed throughout the body.

Although the total copper content in the human body is small, its role is vital. Copper’s involvement in various cellular processes underscores its importance, particularly in cancer research, where its functions and regulation are of significant interest.

### Copper export

Copper is primarily excreted and stored in the liver, highlighting the liver’s crucial role in maintaining copper homeostasis within the body. The ATP7A and ATP7B transporters in the trans-Golgi network (TGN) regulate both the removal and retention of copper ions [31]. Deficiencies in ATP7A and ATP7B can disrupt copper transport, leading to diseases such as Menkes disease and Wilson disease, respectively [32].

In cases of copper overload, ATP7A/B exits the TGN to facilitate the excretion of excess copper ions, along with other unabsorbed metals, through the biliary tract into bile or stool. Conversely, when copper levels are insufficient, ATP7A/B transports copper ions from the TGN to retro-Golgi vesicles. These copper-containing vesicles then move to the plasma membrane, releasing copper into the cell to restore and maintain appropriate copper concentrations. Interestingly, this mechanism is also employed by cancer cells to transport copper ions outside the cell, contributing to the regulation of copper homeostasis within the tumor environment [33]. Thus, maintaining proper copper levels in the body requires a multi-faceted regulatory system that ensures a balance between copper retention and excretion to prevent both deficiency and toxicity.

### Cuproptosis-related gene expressions in breast cancer

Numerous studies have demonstrated that elevated concentrations of copper ions in serum are associated with various types of tumors, including breast cancer [34], liver cance [22], lung cance [35], and stomach cancer. Furthermore, increased copper ion levels have been linked to the stage and progression of breast cancer [36]. Recent findings have verified that copper-induced cell death is mediated by protein lipid acylation, underscoring the integral role of copper toxicity in the development of breast cancer and highlighting its potential as a therapeutic target [37].

Copper is involved in the regulation of numerous genes associated with cell death. Tsvetkov et al. identified ten cuproptosis-related genes (CRG) [38] that are closely associated with the cuproptosis metabolic pathway. These genes can be categorized into seven positive regulatory genes, further divided into three groups: (1) FDX1; (2) Lipoic acid (LA) pathway-related genes: LIAS and LIPT1; (3) Genes encoding components of the pyruvate dehydrogenase complex (PDC), which plays a crucial role in mitochondrial respiration: DLAT, DLD, PDHA1, and PDHB [36, 37].The remaining three genes are negative regulatory genes that, when knocked out, increase sensitivity to copper toxicity: MTF1, GLS, and CDKN2A [39, 40].

**Fig. 3:**
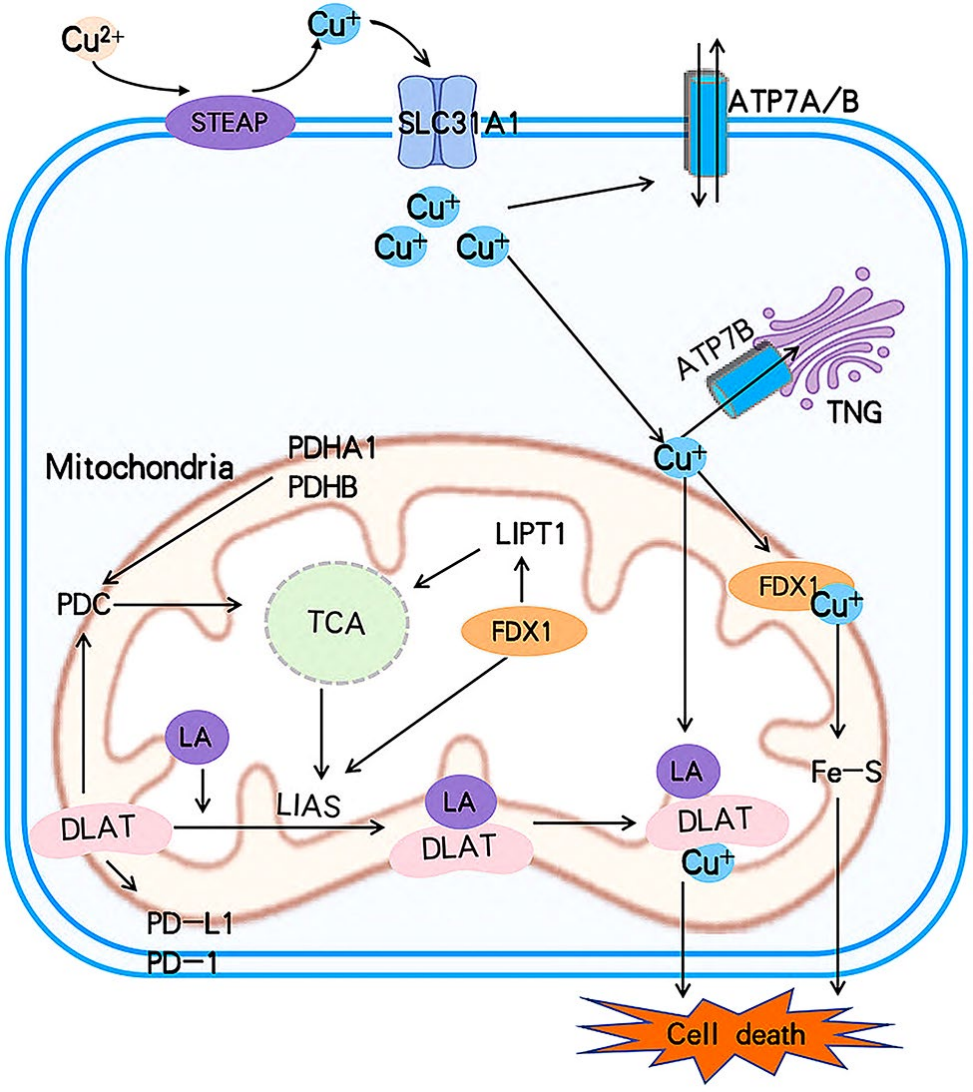
Schematic of cuproptosis mechanism. Cuproptosis can be induced by the increase of intracellular free copper ion, which occurs through four modes of copper absorption, output and storage. SLC31A1 is a copper penetrase that is specific to Cu^+^. Inhibiting ATP7B can reduce copper exports. Excess Cu^+^ binds to the lipified DLAT and subsequently leads to oligomerization of DLAT. Cuproptosis is caused by Copper-mediated reduction of Fe-S stability

The relationship between cuproptosis and cancer remains an active area of research, with studies showing that these genes are strongly associated with tumor prognosis. Table 1 outlines the functions of cuproptosis-related genes and their clinical significance in breast cancer. Cuproptosis is believed to interact with components of the tricarboxylic acid (TCA) cycle in mitochondria and is involved in the conserved post-translational protein modification pathway known as lipoacylation [2]. A schematic representation of this mechanism is provided in Fig. 3.

**Table 1:** The functions of cuproptosis-related genes and their clinical value in breast cance

Gene	Full name	Subcellular locations	Functios	Role in cuproptosis	Clinical values	Ref.
FDX1	Ferredoxin 1	Mitochondrion matrix	The biosynthesis of Fe-S clusters involves the reduction of Cu^2+^ to Cu^+^, which is crucial for the synthesis of various steroid hormones. Additionally, it serves as an electron transport intermediate for mitochondrial cytochromes P450.	FDX1 plays a core role as an upstream regulator of protein acylation in the LA pathway	FDX1 has prognostic value for survival in certain cancer patients such as ACC, KIRC, HNSC, THCA, and LGG.	[41]
LIAS	Lipoic acid synthetase	Mitochondrion	Participating in the synthesis of mitochondrial-related metabolic enzymes, energy metabolism, and antioxidant reactions	Downstream gene regulated by FDX1	High expression of LIAS resulted in poorer overall survival (OS) and progression-free survival (FP), and was associated with more advanced stages of lung cancer.	[42]
LIPT1	Lipoyltransferase 1	Mitochondrion	Regulating glutamine metabolism by catalyzing acyl transfer	Regulated by FDX1 and participating in lipid acylation of DLAT	Elevated expression of LIPT1 was associated with a more favorable prognosis.	[43]
DLAT	Drolipoamide S-acetyltransferase	Mitochondrion matrix	Components of pyruvate dehydrogenase complex	Key enzyme involved in the TCA cycle	An abundance of Cu^+^ binds to lipoylated DLAT, initiating its oligomerization. Ultimately, this chain of reactions leads to cuproptosis.	[35]
PDHA1	Pyruvate dehydrogenase El subnit alpha 1	Mitochondrion matrix	Components of pyruvate dehydrogenase complex	Key enzyme involved in the TCA cycle	NA	
CDKN2A	Cyclin-dependent kinase inhibitor 2 A	Nucleus	Inducing cell cycle arrest in GI and G2 phases	Knocking out lead to sensitive of cuproptosis	Higher expression of CDKN2A conferred risk for lung adenocarcinoma (LUAD) but exhibited a protective function in BRCA.	[44]
SLC31A1	Solute Carrier Family 31 Member 1	Cell membrane	High-afnity, saturable copper transporter involved in dietary copper uptake	overactivaiton lead to intracellular copper accumulation	Alleles of SLC31A1 were associated with a poorer prognosis in both LUAD and BRCA.	[35]
ATP7A	ATPase Copper Transporting Alpha	Cell membrane, trans-Golgi network membrane, plasma membrane	ATP-driven copper ion pump	knock out lead to intracellular copper accumulation	NA	
ATP7B	ATPase Copper Transporting Beta	Cell membrane, trans-Golgi network membrane, plasma membrane	ATP-driven copper ion pump	knock out lead to intracellular copper accumulation	Alleles of ATP7B were linked to a reduced risk of LUAD.	[35]

FDX1, lactate pathway-related genes (LIAS and LIPT1), mitochondrial respiratory key genes (DLAT and PDHA1), and genes that increase sensitivity to cuproptosis by knockdown (CDKN2A)

### FDX1

FDX1, a member of the ferredoxin family of proteins, is primarily located in the mitochondria and serves multiple physiological functions, including mitochondrial respiration, bile acid metabolism, steroid hormone synthesis, and the transformation of cytochrome during vitamin D metabolism [45, 46]. FDX1 plays a critical role in converting Cu^2+^ into the more cytotoxic form, Cu^+^, thereby promoting cellular copper poisoning. This process positions FDX1 as a key mediator in copper-induced cytotoxicity [47].

As an upstream regulator of protein acylation, FDX1 is essential for this modification to occur. When FDX1 is knocked out, protein acylation is lost, thereby shielding cells from the toxic effects of copper poisoning. During copper poisoning, FDX1, as an upstream regulator of the LA pathway, is closely associated with the regulation of the protein lipid acylation process [2].

Studies have shown that FDX1 expression is down-regulated in breast cancer [41]. Additionally, FDX1 expression has been positively correlated with the presence of most cancer immune cells. It is also associated with major histocompatibility complexes, immune suppression, immune activation, chemokines, and chemotaxis [48]. Notably, elevated levels of FDX1 expression have been linked to improved overall survival and progression-free survival in breast cancer patients [49].

Moreover, FDX1 may act as a risk factor for cancer through its regulation of DNA and RNA methylation, mismatch repair (MMR) gene expression, and tumor mutation burden (TMB) [50]. These findings underscore FDX1’s multifunctional role in various biological processes, and its involvement in copper poisoning suggests that it may be a promising therapeutic target in the treatment and progression of breast cancer.

### LIAS

Lipoic acid synthase (LIAS) is a mitochondrial protein essential for the synthesis of mitochondrial metabolic enzymes and plays a pivotal role in antioxidant reactions and energy metabolism [51, 52]. LIAS is also regulated as part of the copper-induced cell death pathway, with its activity necessary for the final biosynthesis of lipoic acid. Deficiencies in LIAS can result in various disorders, including mitochondrial energy metabolism defects, neonatal epilepsy, atherosclerosis, and elevated glycine levels [53].

Studies have demonstrated that the absence of the LIAS gene leads to the accumulation of pyruvate and α-ketoglutarate, a decrease in protein lipoacylation in *C. elegans*, and inhibition of copper-induced cell death [54]. Recent research into the relationship between LIAS and breast cancer (BRCA) has shown that high expression levels of LIAS are associated with improved survival outcomes in breast cancer patients. Notably, an inverse relationship between cytotoxic T-cell infiltration and LIAS expression was observed in patients undergoing anti-CTLA-4 and anti-PD-L1 therapies, suggesting that LIAS gene expression levels could serve as a predictor for the efficacy of immunotherapy in BRCA patients [51]. These findings suggest that LIAS may serve as a valuable prognostic marker for breast cancer, offering insights into patient outcomes and the potential effectiveness of certain therapies.

### LIPT1

Lipoacyltransferase 1 (LIPT1), a member of the lipoacyltransferase family, plays a critical role in regulating several cellular processes, including amino acid metabolism, glycolysis, the tricarboxylic acid (TCA) cycle, and fatty acid production [55, 56]. It is responsible for modulating lipoic acid transport within the TCA cycle and influences mitochondrial metabolism in tumor cells, thereby contributing to cancer cell proliferation, metastasis, and invasion. Notably, when LIPT1 is knocked out, cancer cell growth and invasive capabilities are significantly impaired [57, 58].

Interestingly, LIPT1, a protein associated with copper-induced cytotoxicity, shows lower expression levels in breast cancer tissues compared to normal tissues [59]. LIPT1 is regulated by FDX1 during copper poisoning and is involved in the lipid acylation of DLAT. Given its distinct expression patterns in breast cancer, LIPT1 may serve as a potential biomarker for immunoassays and could provide valuable insights into the disease’s progression and treatment.

### DLAT

Dihydrolipoamide S-acetyltransferase (DLAT), a key component of the pyruvate dehydrogenase complex (PDC), plays a critical role in regulating the energy supply of tumor cells by modulating the citric acid cycle and oxidative phosphorylation pathway [60]. Upon oligomerization, DLAT becomes susceptible to copper-induced toxic stress, leading to cell death. This highlights its significance in the process of copper poisoning and its potential role in cancer treatment [61].

DLAT expression levels vary across different cancer types. For instance, DLAT expression is notably elevated in gastric cancer, suggesting its potential as a therapeutic target. Furthermore, recent studies have shown that DLAT expression is high in patients who exhibit resistance to anti-PD-L1/PD-1 treatments, implying that DLAT could serve as a predictive marker for breast cancer (BRCA) resistance to immunotherapy [62].

### PDHA1

Pyruvate dehydrogenase E1 subunit alpha 1 (PDHA1) and pyruvate dehydrogenase E1 subunit beta (PDHB) are essential components of the pyruvate dehydrogenase complex (PDC), a critical mitochondrial enzyme complex that plays a central role in the tricarboxylic acid (TCA) cycle [60]. PDHA1 may contribute to the regulation of copper-induced cytotoxicity by participating in key processes within the TCA cycle and energy metabolism. PDHA1 expression has been linked to cancer progression and metastasis [63]. Notably, PDHA1 expression levels in breast cancer tissue are lower than in normal breast tissue, and its expression has been associated with breast cancer prognosis [64].

Furthermore, the oncoprotein Hepatitis B X-interacting protein (HBXIP) has been found to enhance glucose metabolic reprogramming in breast cancer by inhibiting cytochrome C oxidase (SCO2) and PDHA1 [65]. In gastric cancer, increased PDHA1 expression is associated with a poor prognosis, while downregulation of this gene promotes cancer progression [66]. Additionally, PDHA1 is closely linked to the growth of prostate cancer and has potential as a gene therapy target for this disease [67]. Given its diverse functions in multiple tumors, PDHA1 represents an important gene for future research, warranting further investigation as a therapeutic target.

### CDKN2A

Cyclin-dependent kinase inhibitor 2 A (CDKN2A), which encodes the p16INK4a protein, is a well-established tumor suppressor gene. Its expression is closely linked to cancer recurrence, poor prognosis, and metastasis [68]. CDKN2A induces cell cycle arrest in the G1 and G2 phases, thereby inhibiting cell proliferation and enhancing the sensitivity of breast cancer cells to chemotherapy [69]. Mutations in CDKN2A can lead to the loss of growth control in several cancers, including BRCA, ovarian cancer (OC), and head and neck squamous cell carcinoma (HNSC) [70, 71].

Research has shown that triple-negative breast cancer (TNBC) patients with elevated CDKN2A expression exhibit higher immunogenicity and may benefit from immunotherapy. Additionally, CDKN2A is associated with epidermal growth factor receptor (EGFR) activity, suggesting its potential as a predictive biomarker for TNBC anti-EGFR therapy [72]. Moreover, overexpression of CDKN2A enhances ATPase activity in mitochondria, depletes copper ions, and prevents copper toxicity caused by elevated copper concentrations [73]. Therefore, regulating CDKN2A expression may represent a promising strategy for preventing and treating BRCA-related copper poisoning.

### SLC31A1

Solute Carrier Family 31 Member 1 (SLC31A1), also known as CTR1, is a key protein involved in copper transport into cells, thereby playing a crucial role in maintaining intracellular copper homeostasis [74]. Research has shown that SLC31A1 expression is significantly elevated in breast cancer (BRCA), correlating with a poorer overall survival rate. Data from the Human Protein Atlas (HPA) further demonstrate that SLC31A1 expression is notably lower in normal breast tissue compared to breast cancer tissue. Additionally, analysis using TIMER reveals a positive correlation between SLC31A1 expression and the presence of immune cells such as CD4 T cells, myeloid dendritic cells, neutrophils, and macrophages.

Notably, high expression of SLC31A1 enhances breast cancer’s sensitivity to porphyrosol, thereby improving its therapeutic effect [75]. Studies also suggest that methylation of the SLC31A1 promoter may influence tumor transcription, contributing to cancer progression [76]. By increasing SLC31A1 expression on the cell membrane, the uptake of copper ions by cancer cells is enhanced, promoting copper-induced cytotoxicity, or “copper poisoning,” which ultimately exerts an anti-cancer effect [77]. Consequently, SLC31A1 presents an intriguing target for breast cancer treatment strategies.

### ATP7A and ATP7B

ATPase Copper Transporting Alpha (ATP7A) and ATPase Copper Transporting Beta (ATP7B) are essential copper transporters responsible for regulating the transport of copper from intracellular to extracellular spaces, thus maintaining copper homeostasis. These transporters occupy two distinct cellular locations: one in the trans-Golgi network (TGN), where Cu-ATPase supplies copper cofactors to cuproenzymes, and the other in vesicles or the plasma membrane, where Cu-ATPase eliminates excess copper [78].

Nagaraja et al. demonstrated that the expression level of ATP7A in invasive breast cancer tissues exceeds that in non-invasive breast cancer tissues [79]. Similarly, ATP7B expression is elevated in several cancers, including ovarian, stomach, esophageal, liver, breast, and oral squamous cell carcinomas [80].The altered expression levels of ATP7A and ATP7B may disrupt copper homeostasis in breast cancer cells, making them potential targets for therapeutic intervention in breast cancer treatment.

### Potential therapeutic targets and signaling pathways of cuproptosis in breast cancer

**Fig. 4:**
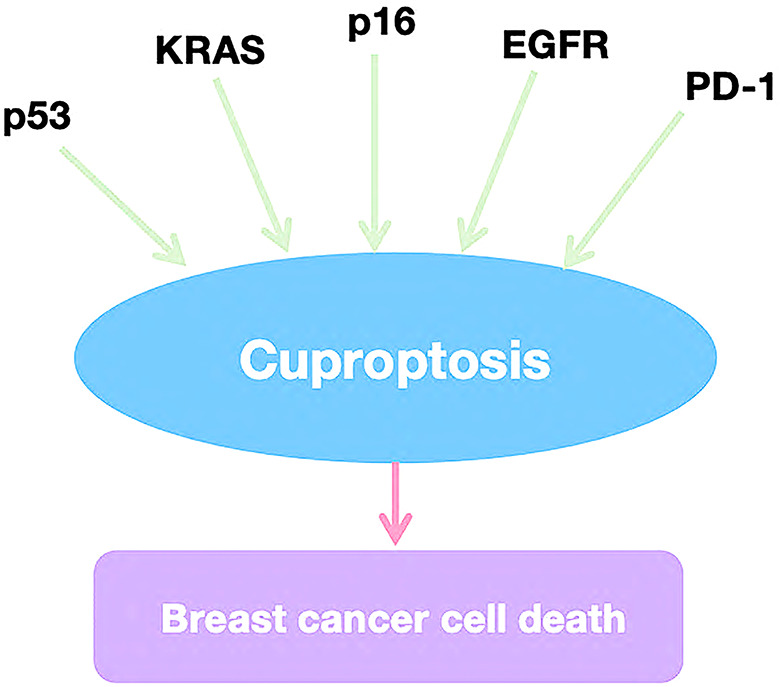
Potential therapeutic targets and signaling pathways for cuproptosis in breast cancer

In recent years, tumor-targeting therapies have achieved significant success in breast cancer treatment, making the exploration of tumor-related signaling pathways and therapeutic targets a focal point of research. Copper-induced cell death (cuproptosis) has been linked to several key signaling pathways and targets, including PD-L1, KRAS, and EGFR. Understanding these mechanisms provides valuable insights for applying cuproptosis in the clinical management of breast cancer (Fig. 4).

### KRAS

KRAS plays a critical role in the development of breast cancer and is associated with genetic susceptibility to the disease. A study has identified mutations in the rs9266 gene locus within KRAS, which may be linked to an elevated risk of breast cancer in women [81]. Additionally, the tricarboxylic acid cycle (TCA) and reactive oxygen species (ROS) generated in mitochondria are essential for KRAS-induced growth, highlighting the importance of mitochondrial metabolism in KRAS-mediated cell proliferation and tumorigenesis [82]. Targeted inhibition of glutaminase (GLS) has been shown to synergistically enhance the anti-tumor activity of selumetinib in KRAS-mutant non-small cell lung cancer (NSCLC), suggesting that copper metabolism-related pathways could offer novel therapeutic strategies for targeting KRAS in breast cancer [83].

### p53

The tumor suppressor protein p53 is closely linked to the regulation of breast cancer cell proliferation. Notably, the overexpression of ZNF500 has been shown to inhibit breast cancer cell proliferation both in vivo and in vitro by activating the P53-P21-E2F4 signal transduction axis and directly interacting with p53 via its C2H2 domain [84]. Although no direct connection between p53 and copper-induced cell death (cuproptosis) has been established, it is known that p53 activation preserves mitochondrial integrity and function and participates in regulated cell death (RCD) by targeting apoptosis-inducing factor (AIF) [7, 85]. These findings suggest that p53 may indirectly promote copper-induced cell death by supporting mitochondrial metabolism.

### EGFR

Mutations in the epidermal growth factor receptor (EGFR) are known to elicit significant tumor responses. Stanniocalcin 1 (STC1) has been found to facilitate lung metastasis in breast cancer by enhancing EGFR and ERK phosphorylation, as well as upregulating the expression of S100 calcium-binding protein A4 (S100A4) [86]. Interestingly, EGFR can also be activated by copper ions even in the absence of its ligands, indicating a significant relationship between EGFR and copper poisoning. This discovery suggests a novel therapeutic approach, whereby the interaction between EGFR and copper can be explored further in breast cancer treatment.

### PD-1

The programmed cell death-1 receptor (PD-1) is an immune checkpoint inhibitor found on the surface of immune effector cells. Its activation is primarily mediated by PD-L1, which can be expressed by a wide range of human cells. Numerous studies have demonstrated the efficacy of blocking PD-1 or PD-L1 with specific antibodies, especially in breast cancer, where metastatic triple-negative breast cancer (TNBC) has shown promising responses [87]. Notably, Voli et al. reported that copper-chelating agents, which induce copper poisoning, can enhance the therapeutic efficacy of anti-PD-L1 treatments. This suggests that the impact of copper poisoning on immune checkpoint suppression may improve the overall anti-tumor effect of these therapies [88].

### p16

The overexpression of the tumor suppressor protein p16 has been associated with estrogen receptor-positive breast cancer cell lines and a diminished response to CDK4/6 inhibitors in breast cancer patients [89]. CDKN2A, the gene encoding both p16INK4a and p14ARF, plays a critical role in cell cycle regulation [90]. Importantly, the knockout of CDKN2A leads to increased sensitivity of cancer cells to copper-induced cytotoxicity. This finding suggests that exploring the relationship between p16 and cuproptosis could offer a novel therapeutic approach for the treatment of breast cancer.

### Cuproptosis therapeutic strategy in breast cancer

The discovery of cuproptosis has opened new therapeutic possibilities for breast cancer treatment. Numerous potential drugs, including copper compounds and combination therapies, along with nanotechnology-based methods, have provided a solid foundation for targeting breast cancer. Cuproptosis occurs primarily within mitochondria and is closely associated with the tricarboxylic acid (TCA) cycle. This suggests that breast cancer cells with heightened mitochondrial metabolism may be more vulnerable to copper ionophores and copper complexes, which form the basis for copper-induced cell death [91]. Overall, cuproptosis represents a promising approach to overcoming the challenges of breast cancer, potentially leading to significant advancements in clinical treatment.

Cu ionophores, defined as specific compounds or chemicals, have the ability to bind to Cu and transport it into cells, thereby elevating the intracellular Cu levels (Table 2). Elesclomol is a bis(thiohydrazide) amide compound capable of chelating extracellular Cu2 + in a 1:1 ratio, resulting in the formation of an elesclomol-Cu2 + complex which facilitates the transport of copper into the cell [92, 93]. For decades, elesclomol has been recognized for its anti-tumor activity, which was presumed to be copper-dependent. Additionally, Tsvetkov et al. observed that elesclomol-Cu can induce cell death via the cuproptosis pathway [2]. Elesclomol-Cu can eliminate tumor cells by elevating the level of reactive oxygen species (ROS) in cancer cells [94–96], further indicating that elesclomol-Cu possesses multiple mechanisms for killing tumor cells.Furthermore, recent research has unveiled the potential therapeutic benefits of elesclomol in addressing copper deficiency. Notably, elesclomol-facilitated copper delivery to mitochondrial cuprin persists even in the absence of FDX1, highlighting a distinctive mechanism of intracellular copper transport mediated by elesclomol [97]. Whether non-mitochondrial copper plays a role in cell death pathways other than cuproptosis induced by elesclomol-Cu remains an area requiring further exploration. The intricate interplay between mitochondrial metabolism, elesclomol, and cuproptosis is currently not well understood, and additional research is crucial to elucidate this mechanism. Such insights could potentially aid in the development of Elesclomol-Cu-based anticancer strategies. Notably, elesclomol-Cu has yet to demonstrate any therapeutic efficacy in clinical trials [54]. The insufficient elevation of copper levels in cancer cells by monotherapy with elesclomol may account for the failure to induce cuproptosis. Findings from a Phase III clinical trial demonstrated that the combination of elesclomol and paclitaxel did not yield significant improvements in melanoma patients; however, elesclomol exhibited enhanced antitumor effects specifically in patients with low lactate dehydrogenase (LDH) levels [98]. Low LDH levels indicate reduced glycolysis [99] and heightened mitochondrial metabolism, which aligns with Tsvetkov’s discovery that cuproptosis is reliant on mitochondrial metabolism.

Disulfiram(DSF), an aldehyde dehydrogenase (ALDH) inhibitor, has been approved by the FDA for the treatment of alcoholism. Additionally, it has undergone extensive research over a prolonged period in the field of anti-tumor studies (Table 2).DSF interacts with copper as a copper ionophore, resulting in the formation of the metabolite bisdiethyldithiocarbamate-Cu (CuET) [100]. This metabolite facilitates the transport of copper across the cell membrane. Cellular damage induced by Disulfram-Cu is also associated with apoptosis, ferroptosis, and cuproptosis. Several targets or signaling pathways have been reported to be linked to the anti-tumor activity of Disulfram-Cu, like ROS levels [101], the ubiquitin–proteasome system [12], the NF-kB pathway [102], and NPL4 [103]. Furthermore, disulfram-Cu has been reported to possess the ability to overcome tumor drug resistance to cisplatin [104] and paclitaxel [101, 105]. Table 1 summarizes the anti-tumor function of disulfram in preclinical studies. Although disulfram, similar to elesclomol, has demonstrated significant anti-tumor effects in preclinical experiments, promising results from clinical trials are still awaited [106]. As previously mentioned, maintaining high copper levels in the patient’s cancer cells is challenging. However, considering the favorable clinical safety profile, conducting additional combined clinical trials and integrating elesclomol or disulfram with existing clinical drugs may pave the way for the translation of cuproptosis-associated anticancer therapy from the laboratory setting into clinical practice.

**Table 2:** The anti-breast cancer effect of copper ionophores

Compound	Cancer type	Materials (cell lines)	Combined drugs	Anti-tumor efect and its involved mechanism	Refs.
Elesclomo	Breast cancer	MCF-7, MDA-MB-231, HCC1806	DOX, paclitaxel	Elesclomol moderately curbs breast cancer cell growth and boosts DOX/paclitaxel-induced apoptosis, partly through the JNK1 pathway	[107]
Elesclomol	Breast cancer	BRCA1-mutated breast cancers cells, Basal-like breast cancers cells		BRCA1-mutated and/or basal-like breast cancer cells sensitive to elesclomol due to defective oxidative DNA repair	[108]
Disulfram	Breast cancer	MCF-7, SKB-R3, MDA-MB-435 S	Cisplatin	Disulfram curbs ALDH, reduces breast cancer stemness, and boosts cisplatin toxicity	[104]
Disulfram	Breast cancer	MCF-7, MDA-MB-231		Disulfram blocks TGF-β-induced EMT in breast cancer via ERK/NF-κB/Snail downregulation	[102]
Disulfram	Breast cancer	LLC, B16		Disulfram halts FROUNT-chemokine interaction, inhibiting macrophages and reducing tumor progression	[109]
Disulfram	Breast cancer	BT-549, MDA-MB-231		Disulfram boosts anti-PD-1 therapy in TNBC by epigenetic IRF7 reactivation, modulating PD-L1	[110]
Disulfram	Breast cancer	MCF-7	DOX, hydrazine	The triple therapy of DOX, disulfram, hydrazine boosts chemosensitivity by synergistically reducing DOX dose for eradicating resistant MCF-7 cells	[111]
Disulfram/Cu	Breast cancer	MCF-7, MDA-MB-231		CuET inhibits NPL4/p97-mediated protein degradation, suppressing tumor growth	[103]
Disulfram/Cu	Breast cancer	MCF7, MDA-MB-231, T47D	Paclitaxel	Disulfram–Cu halts breast cancer stem cell growth, boosts paclitaxel cytotoxicity in breast cancer cells, likely via ROS induction and NF-κB inhibition	[101]
Disulfram/Cu	TNBC	MDA-MB-231PAC10	Paclitaxel/cisplatin	Disulfram–Cu inhibits the expression of cancer stem cell markers and reverses paclitaxel and cisplatin resistance in MDA-MB-231PAC10 cells	[105]
Disulfram/Cu	Breast cancer	MDA-MB-231, MCF10DCIS.com		Disulfram–Cu induces apoptosis and suppresses breast cancer xenograft growth by inhibiting proteasome activity	[12]
Disulfram/Cu	Breast cancer	MDA-MB-231, BT20, MDA-MB-231PIK3CA H1047R, MDA-MB-231PIK3CA-E545K		Disulfram–Cu decreases PTEN expression and AKT activation in breast cancer cells, and when combined with the PI3K inhibitor LY294002, it potently suppresses the growth of xenografts formed by MDA-MB-231 cells harboring mutant PIK3CA-H1047R or PIK3CA-E545K	[112]
Disulfram/Cu	TNBC	MDA-MB-231, 4T1		Disulfram–Cu treatment triggers apoptosis in TNBC cells through caspase-3 activation and selectively targets cancer stem cell-like populations, with these effects attributed to significant disruption of the STAT3 signaling pathway	[113]
Disulfram/Cu	Breast cancer	BT474, SKBR3		Disulfram–Cu triggers apoptosis and eliminates cancer stem-like cells in HER2-positive breast cancer by inhibiting the HER2/Akt signaling pathway	[114]
Disulfram/cadmium (Cd) Breast cancer	Breast cancer	MCF10DCIS, MCF10A		Disulfram–Cd selectively disrupts proteasome function and induces apoptosis in human breast cancer cells, while leaving non-tumorigenic cells unaffected	[115]
Disulfram/Cu	Breast cancer	MDA-MB-231, Hs578T		Disulfram–Cu exhibits anti-migratory and anti-invasive effects by inducing focal adhesion loss and cytoskeletal collapse, thereby inhibiting tumor growth and lung colonization in triple-negative breast cancer (TNBC)	[116]
Disulfram/Cu	Breast cancer	MCF-7, HT-29		An acidic pH significantly augments the toxicity of the disulfram–Cu complex in breast and colon cancer cells. This effect is correlated with alterations in cell metabolism, modulation of Akt kinase and NF-κB activity, and elevated production of reactive oxygen species (ROS)	[117]

ES elesclomol, DSF disulfram, DOX doxorubicin, ROS reactive oxygen species, GdECs GSC-derived endothelial cells, ARID1A AT-rich interactive domain 1 A, TMZ temozolomide, BRCA1 breast cancer susceptibility gene 1, EMT epithelial-mesenchymal transition, HCC hepatocellular carcinoma, TGF-β transforming growth factor-β, PD-L1 programmed death-ligand 1, PARP poly ADP-ribose polymerase, PTEN Phosphatase and tensin homolog deleted on chromosome ten, CuET bis-diethyldithiocarbamate-Cu, TNBC triple negative breast cancer, JNK Jun N-terminal kinase, HER2 human epidermal growth factor receptor 2

Copper serves as a dual-edged sword in the context of tumorigenesis. Elevated copper levels facilitate tumor cell proliferation and growth, hinting at the existence of resistance mechanisms against cuproptosis within tumor cells. Consequently, small-molecule compounds capable of disrupting copper homeostasis may serve to induce or augment tumor cell sensitivity to cuproptosis(Table 3). For example, Yang et al. revealed that zinc pyrithione can elicit cuproptosis in triple-negative breast cancer (TNBC) cells by disturbing intracellular copper homeostasis and inhibiting DLAT oligomerization, potentially enhancing the chemosensitivity of TNBC [117].Small compounds have the ability to induce cancer cell death without the need for additional copper, thereby circumventing the side effects associated with metal ion imbalance during treatment, unlike copper ionophores. However, research into cuproptosis is still in its nascent stages.The advancement and discovery of further cuproptosis inducers, particularly those derived from drugs already approved for clinical use, have facilitated the clinical application of cuproptosis-targeted breast cancer treatment strategies.

**Table 3:** Small compounds inducing cuproptosis in breast cancer

Compound	Cancer type	Materials (cells)	The efect on cuproptosis and its involved mechanism	Refs.
ZnPT	TNBC	MDA-MB-231, HCC1806	ZnPT induces cuproptosis by disrupting intracellular copper homeostasis and inhibiting DLAT oligomerization. The induction of cuproptosis by ZnPT potentially enhances the chemosensitivity of TNBC cells	[117]

ZnPT zinc pyrithione, TNBC triple negative breast cancer

In recent years, nanosensitizers have gained significant popularity, and numerous research strategies have unveiled their unique physical properties, enabling them to effectively target and eliminate deep-seated malignant tumors. These nanosensitizers are responsive to highly penetrating stimuli, such as those utilized in photothermal therapy (PTT), sonodynamic therapy (SDT) [118–123], X-ray-induced photodynamic therapy (PDT) [124–126], and chemodynamic therapy (CDT), among others. Notably, the application of copper in breast cancer treatment has been extensively reported, particularly in the context of CDT [27, 127–138], PTT [139–146], PDT [147–149], chemotherapy [150–152], or combinations of these therapies [153–155](Table 4).

**Table 4:** Nanomedicines targeting cuproptosis for breast cancer treatment

Nanomaterials composition	Type	Cancer	Tested model	Effects OR Involved mechanism	Ref.
Cu@cLAs	CDT	BC	MCF-7/R nude mice	Cu@cLAs were dissociated into LA and dihydrolipoic acid (DHLA), thereby releasing Cu^2+^ and Cu^+^ ions. This process facilitated the efficient elimination of cancer cells by delaying metabolic depletion and elevating the ROS levels within tumor cells.	[127]
Cu-siMDR-CDDP	CDT	BC	MCF-7/CDDP cells	Upon release from Cu-siMDR-CDDP, CDDP initiates a cascade of bioreactions involving NADPH oxidase (NOX) and superoxide dismutase (SOD) in the acidic tumor microenvironment (TME), resulting in the production of H_2_O_2_. This H_2_O_2_ undergoes a Cu^2+^-catalyzed Fenton-like reaction, converting it into hydroxyl radicals (HO•) and causing a depletion of glutathione (GSH). This depletion disrupts the redox adaptation mechanism of drug-resistant cancer cells. Furthermore, HO•-induced lysosome destruction facilitates the delivery of MDR1 siRNA, which subsequently inhibits the expression of P-glycoprotein (P-gp) and reduces the efflux of CDDP.	[129, 130]
NH2-MIL-101(Fe)/D-pen	CDT	BC	MCF-7 cells	The released d-pen chelated Cu, which is highly abundant in cancer environments, leads to the production of excess H_2_O_2_. This H_2_O_2_ is then decomposed by the Fe present in NH2-MIL-101(Fe), generating hydroxyl radicals •OH. Consequently, the cytotoxicity of NH2-MIL-101(Fe)/d-pen was observed in cancer cells.	[128]
NH2-MIL-101(Fe)/CPT-11	CDT	BC	MCF-7R-bearing BALB/c nude mice	Among all the tested formulations, the combined formulation demonstrated the most significant anticancer effects, attributed to the synergistic interaction between CDT and chemotherapy.	[128]
Cu-Cys NPs	CDT	BC	MCF-7R-bearing NOD SCID mice	In situ glutathione-activated CDT, reinforced by H_2_O_2_, induces tumor cell apoptosis. Cu-Cys NPs effectively inhibited drug-resistant breast cancer in vivo without causing notable systemic toxicity.	[132]
Hollow Cu9S8 NPs	CDT	BC	4T1 tumor-bearing mice	Compared to solid Cu9S8 NPs, the increased number of active sites and enhanced photothermal performance result in enhanced CDT.	[156]
Vk3 @MOF-199	CDT	BC	4T1 tumor-bearing mice	NQO1 catalyzes Vk3 to generate sufficient H_2_O_2_, which amplifies the effect of CDT.	[134]
DOX@BSA-Cu	CDT	BC	4T1 cells and MCF-7 cells	DOX enhances the H_2_O_2_ content and promotes the generation of hydroxyl radicals, thereby amplifying the effectiveness of CDT.	[157]
mCMSN	CDT	BC	MCF-7tumor-bearing mice	Target-cell-specific GSH depletion enhances CDT, while simultaneously relieving hypoxia to improve PDT.	[133]
Au-CuS YSNPs	CDT/PDT/PTT	BC	4T1 tumor-bearing mice	Au-CuS YSNPs enhance the efficacy of PDT/PTT due to their localized surface plasmon resonance effect.	[155]
FA-HMCu2-xS/BLM/LM	CDT/PDT/PTT/CT	BC	MCF-7 tumor-bearing mice	NIR-responsive drug release triggers further activation of BLM, leading to DNA cleavage.	[158]
Dox@Cu-Met NPs	CDT/CT	BC	Breast-tumor-bearing mice	In the TME, dual-stimuli responsive drug release triggered by both pH and GSH levels mutually enhances the efficacy of CT and CDT.	[159]
Cu3BiS3 NCs	PDT	BC	MCF-7 tumor xenograft-bearing mice	Cu3BiS3 nanocrystals achieve complete tumor regression using an ultra-low dose of NIR laser irradiation.	[148]
BP-CuS-FA	PDT/PTT	BC	4T1 tumor-bearing mice	A biocompatible and photodegradable CuS carrier enables a single laser-activated process for both PDT and PTT.	[160]
CuxS/Au-PEG NPs	PTT	BC	EMT-6 tumor-bearing mice	Upon irradiation with a 1064 nm laser, the tumors experience an enhancement in their oxygenation status. Subsequently, the combination of photothermal therapy and radiotherapy yields remarkable synergistic therapeutic effects. This study introduces a novel concept for the design of a new-generation nanomedicine aimed at tumor thermoradiotherapy.	[141, 142]
[(64)Cu]CuS NPs	PTT	BC	BT474 breast tumor	RT/PTT significantly delayed tumor growth in the subcutaneous BT474 breast cancer model and markedly extended the survival of mice harboring orthotopic 4T1 breast tumors. Furthermore, RT/PTT decreased the number of lung tumor nodules and inhibited the formation of tumor mammospheres from treated 4T1 tumors.	[145, 146]
CuCo(O)/GOx@PCNs	PTT/IMT/ST	BC	4T1 tumor-bearing mice	CuCo(O)/GOx@PCNs can achieve oxygen supply, glucose consumption, and photothermal ablation. Additionally, the immune response effect can further suppress tumor metastasis and recurrence.	[27, 135, 136]
MSN-DNA-CuS	PTT/CT	BC	HeLa and MCF-7 cells	Photothermal controllable and GSH-responsive drug release.	[161]
CuPd TNP-1	PTT	BC	4T1;MCF7/MDR	The inhibition of autophagy through the use of 3-methyladenine or chloroquine exhibits a notable synergistic effect when combined with TNP-1-mediated PTT in triple-negative (4T1), drug-resistant (MCF7/MDR), and patient-derived breast cancer models.	[143, 144]
Cyclodextrin DDC-Cu inclusion complexes	CT	BC	MDA-MB-231 cells	Cyclodextrin enhances the solubility of DDC-Cu while also increasing its toxic effect.	[152]
DSF@PVP/Cu-HMPB	CT	BC	4T1 tumor-bearing mice	TME-triggered release of Cu^2+^ facilitates the generation of the in situ anti-cancer complex CuL2, and NIR irradiation further enhances its anti-cancer activity.	[162]

CT, chemotherapy; CDT, chemodynamic therapy; Vk3 @MOF-199, Cu-based metal-organic framework-199 nanoplatform integrating vitamin k3; Cu@cLAs, copper on cross-linked lipoic acid nanoparticles; SOD, superoxide dismutase; PTT, photothermal therapy; NOX, NADPH oxidases

Recent developments in Cu-based nanoagents have leveraged synthetic chemistry and surface modification techniques for selective tumor delivery and effective treatment [163]. Specifically, Cu-based chalcogenide nanoagents, such as Cu₂−χS, Cu₂−χSe, and Cu₂−χTe, have demonstrated superior performance in converting light energy into heat energy within the second near-infrared (NIR-II) window, ranging from 1000 to 1350 nm [164, 165]. These properties make them suitable for photoacoustic imaging (PAI) and photothermal therapy (PTT) of tumors. Inspired by the Warburg effect [166], a novel nanoagent, metabolism-targeting Cu₂−χS (MACuS), was developed using a glucose-mediated biomineralization strategy.

MACuS distinguishes itself from traditional phototherapy agents by combining Fenton-like reactions [167, 168] with photothermal conversion capabilities.A study was conducted to investigate the mechanism by which MACuS catalyzes the production of reactive oxygen species (ROS) [169–172] and its capacity to induce immunogenic cell death (ICD) [173] in copper-based nanoagents. The findings revealed that MACuS-induced cuproptosis gradually increases intra-tumor oxidative stress through ROS production while also reducing glutathione (GSH) levels [174, 175]. Cuproptosis was found to be crucial for triggering ICD, which subsequently leads to immune activation and tumor eradication following treatment with MACuS and/or NIR-II irradiation.Furthermore, the study observed that MACuS regulates the immunosuppressive tumor microenvironment (TME), thereby enhancing systemic anti-tumor immunity and preventing tumor metastasis and recurrence. These distinct advantages position MACuS as a superior alternative to traditional light therapeutics and nanomedicine [176].

Currently, breast cancer patients may encounter therapeutic drug resistance, rendering drug treatment ineffective and potentially causing tumor progression or recurrence. Recent research indicates that copper transport mechanisms could play a pivotal role in mediating drug resistance in cancer [177]. A growing number of copper complex-based drugs have demonstrated cytotoxicity specifically towards resistant cancer cells. Table 5 outlines the latest advancements in complex-based drugs as promising chemotherapy agents for breast cancer. Beyond a dozen additional platinum compounds, several other metal-based complexes, including those derived from copper, have entered clinical trials in recent decades [178].

**Table 5:** Overcoming cancer drug resistance using copper-based approaches

Compound	Cancer type	Resistance	Effects Or involved mechanism	Ref.
Cu₂−χTe Nanocubes	BC	Multi-Drug Resistant MDA MB 453	In this study, we present our findings on eliciting a response from a specific cancer lineage to chemotherapy through the utilization of multifunctional copper telluride (Cu₂−χTe) nanocubes (NCs) as both photothermal and photodynamic agents, resulting in notable anticancer efficacy.	[179]
CD NPs	BC	MDA-MB 231 cells	CD NPs exhibited greater potential in inducing apoptosis, inhibiting the hypoxia-inducing factor gene, and eliminating CD44 + CSCs by downregulating their stemness, chemoresistance, and metastatic genes, while also reducing the hepatic tumor marker (α-fetoprotein).	[180]
DSF/Cu	TNBC	Taxol	DSF/BKM120 enhanced the antitumor activity of Taxol and postponed tumor recurrence in vivo.	[181]

TNBC, triplenegative breast cancer; BC, breast cancer

Curcumin and its derivatives have been reported to exert certain effects on triple-negative breast cancer (TNBC) cell lines in vitro. Additionally, Fe3+-curcumin (Fe-Cur₃) and Cu2+-curcumin (CD) compounds have been synthesized, with CD being encapsulated in a poly(styrene)-co-maleic acid (SMA) micelle to enhance its stability. In vivo studies have shown that SMA-CD exhibits dose-dependent cytotoxicity, which can enhance the anticancer effect and inhibit the progression of TNBC, without significant adverse effects in a TNBC mouse model [182]. Therefore, the curcumin-copper complex provides a reliable foundation for the direction of breast cancer treatment.

Additionally, the ternary copper complex consists of 1,10-phenanthroline and tyrosine [Cu(phen)(L-tyr)Cl]·3H2O. These two substances are used to cleave DNA while inhibiting the protein degradation system (proteasome), making this copper complex a dual-target compound. [Cu(phen)(L-tyr)Cl]·3H2O may induce apoptosis and cycle arrest in MCF-7 and MDA-MB-231 breast cancer cells by regulating p53, Bax, caspase-9, caspase-3, and caspase-7. The copper complex holds great potential for the treatment of breast cancer [183].

### Conclusions and future prospects

Despite significant advancements in cancer treatment, breast cancer remains a major public health challenge. Due to various lifestyle and environmental factors, the incidence of breast cancer continues to rise, with no substantial improvements in reducing its prevalence. As a result, there is an urgent need to identify novel therapeutic targets and explore new strategies for breast cancer treatment.

Copper is an essential metal for human health, but its accumulation can lead to copper-induced cell death, or cuproptosis. This process is closely associated with mitochondria, the primary organelles responsible for energy production. The link between copper toxicity and mitochondrial function presents a promising therapeutic approach for treating breast cancer. However, several challenges remain. One of the primary limitations of cuproptosis-based therapies is the non-targeted delivery of copper ions. Future research should focus on developing strategies to enhance specificity and minimize off-target effects. This could involve directly targeting intracellular mechanisms involved in cuproptosis, exploring cuproptosis-related gene therapies, and utilizing innovative approaches, such as nanotechnology-based drugs, to deliver copper ionophores to specific breast cancer cell populations or even subcellular compartments.

In the future, combining cuproptosis-targeting therapies with traditional treatment options may provide a comprehensive and effective solution for breast cancer. These combined approaches could offer a more precise, efficient, and personalized treatment plan, leading to better patient outcomes and new advancements in cancer therapy.

## Data Availability

No datasets were generated or analysed during the current study.
